# Data-Driven Extraction of Human Kinase-Substrate Relationships From Omics Datasets

**DOI:** 10.1016/j.mcpro.2025.100994

**Published:** 2025-05-15

**Authors:** Benjamin Dominik Maier, Borgthor Petursson, Alessandro Lussana, Evangelia Petsalaki

**Affiliations:** European Molecular Biology Laboratory, European Bioinformatics Institute (EMBL-EBI), Wellcome Genome Campus, Hinxton, Cambridgeshire, United Kingdom

**Keywords:** kinase-substrate prediction, signaling networks, machine learning, web server

## Abstract

Phosphorylation forms an important part of the signaling system that cells use for decision making and regulation of processes such as cell division and differentiation. In human, >90% of identified phosphosites do not have annotations regarding the relevant upstream kinase. At the same time around 30% of kinases (as annotated in UniProt) have no known target. This knowledge gap stresses the need to make large-scale, data-driven computational predictions. In this study, we have created a machine learning-based model to derive a probabilistic kinase-substrate network from omics datasets. Our methodology displays improved performance compared to other state-of-the-art kinase-substrate prediction methods and provides predictions for more kinases. Importantly, it better captures new experimentally identified kinase-substrate relationships. It can therefore allow the improved prioritization of kinase–substrate pairs for illuminating the dark human cell signaling space. Our model is integrated into a web server, SELPHI_2.0_, to allow unbiased analysis of phosphoproteomics data, facilitating the design of downstream experiments to uncover mechanisms of signal transduction across conditions and cellular contexts.

## Highlights

• SELPHI_2.0_ uses machine learning to predict kinase–substrate interactions.
• Our model outperforms others and expands predictions to additional kinases.
• SELPHI_2.0_ aids hypothesis generation for novel kinases and phosphosites.
• A web server enables analysis of phosphoproteomics across conditions.
• We offer a probabilistic network for context-specific signaling inference.

## Graphical Abstract

**Figure:**
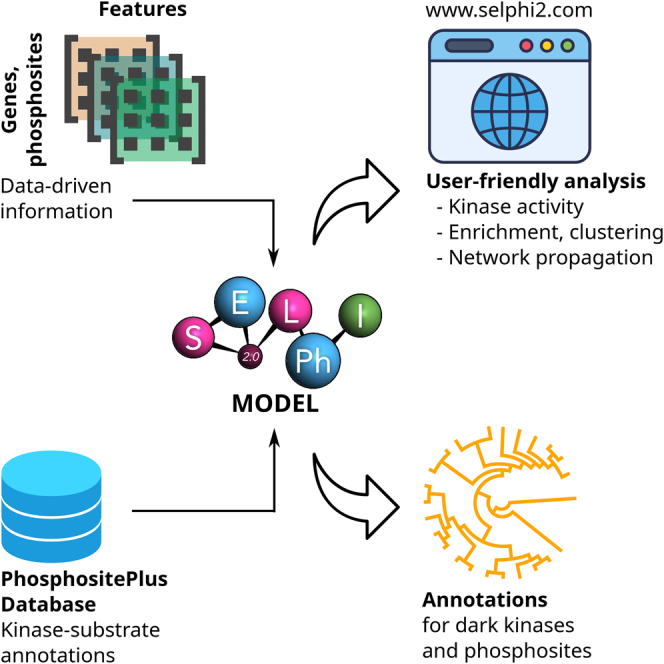

## In Brief

Kinase signaling is central to cellular regulation, yet the majority of phosphosites lack known upstream kinases, and existing pathway databases fail to capture condition-specific dynamics. We present SELPHI_2.0_, a machine learning framework that predicts kinase-substrate interactions at the phosphosite level. SELPHI_2.0_ outperforms existing methods, extends predictions to understudied kinases, and enables functional hypothesis generation. To facilitate its use, we developed a web server that extracts context-specific signaling networks from phosphoproteomics data, providing a more accurate alternative to literature-derived pathways.

Cells communicate through signaling networks (1), which are primarily regulated by post-translational modifications (PTMs). Among these, phosphorylation is the most extensively studied (2) and is catalyzed by kinases that modify specific substrates, altering their state and/or function. The significance of understanding kinase regulatory networks is underscored by the fact that many FDA-approved drugs and emerging targeted therapies focus on kinases (3).

Despite this, only ∼5% of the 100,000+ known phosphorylation sites have an identified upstream kinase to date (4). Furthermore, ∼150 kinases lack known substrates, and ∼80% of annotated phosphosites are attributed to just 20% of the kinases. Notably, studies have shown that understudied kinases can be just as critical for health as well-characterized ones (4), revealing a bias in the literature that favors well-established proteins, possibly due to the fact that they are easier to detect or study. Additionally, publicly available databases such as KEGG (5), Reactome (6), and Omnipath (7) provide a static representation of signaling pathways, reflecting an "average" cell rather than capturing condition-specific dynamics. Consequently, these literature-derived pathways offer limited explanatory power when interpreting phosphoproteomics data.

Mass spectrometry-based phosphoproteomics provides a relatively unbiased view of a cell’s signaling state and has the potential to describe condition-specific signaling networks (8, 9). However, most high-performing methods for network inference rely on prior knowledge, typically derived from established pathways (8, 9), thereby reinforcing existing literature biases.

For example, PropheticGranger, one of the top-performing methods in the HPN-DREAM challenge (8, 9), applies heat diffusion combined with L1-penalized regression (10) on a network built from the Pathway Commons database (11). This reliance on predefined networks underscores the need for more unbiased priors to improve the inference of signaling networks from phosphoproteomics data.

Several methods have been developed to predict kinase–substrate interactions (12), using various approaches, including kinase specificity maps in the form of Position-Specific Scoring Matrices (PSSMs) (13, 14, 15, 16), protein interaction networks (17), structural information (18), or combinations of these (19). More recent strategies incorporate co-expression at the protein level (20) or co-phosphorylation patterns (21).

These methods employ a range of computational techniques, from basic PSSM scoring and log-odds ratios (14) to more advanced models such as profile Hidden Markov Models (HMMs) (16), machine learning (neural networks (19), SVMs (22), Bayesian decision theory (23)), and more recently, deep learning and transfer learning (24, 25). However, most approaches can only predict interactions for a limited subset of the kinome, with many restricted to kinase family-level predictions rather than individual kinases.

Recently, PSSMs describing the specificity preferences of nearly all human kinases have become available (26, 27). While this resource is invaluable for mapping kinase-substrate relationships, it remains challenging to distinguish kinases with similar specificity profiles. Integrating co-expression data (at both gene and protein levels) alongside other biological information can help resolve these ambiguities, enabling the data-driven generation of kinase-substrate regulatory networks.

In our previous work, we developed a machine learning approach that integrates multiple predictors—including co-phosphorylation, co-expression, and kinase specificity models—to construct a data-driven kinase–kinase regulatory network (28). However, this network was limited to predictions at the protein level, whereas individual proteins often have multiple functional phosphosites, which can have distinct or even opposing roles. For example, phosphorylation of Y530 on Src kinase inhibits its activity, whereas dephosphorylation of Y530 and phosphorylation of Y419 activate it (29).

Moreover, while kinase regulatory networks form the backbone of phospho-signaling, non-kinase substrates also play crucial roles. Examples include adaptor proteins that function as scaffolds for signal propagation (30), phosphatases that remove phosphosites to terminate signaling, and transcription factors regulated through phosphorylation to control their activity and translocation (31).

To address these limitations, we extended our previous machine learning model by incorporating additional predictors to include non-kinase substrates and identify kinase-substrate relationships at the phosphosite level. By integrating our predictions with experimentally corroborated kinase–substrate interactions, we identified 76 high-confidence interactions (27, 32, 33), and showcase the use of our resource for functional hypotheses generation for understudied kinases. Our method, SELPHI_2.0_ (Systematic Extraction of Linked PHospho-Interactions 2.0), enables predictions for less-studied kinases and substrates, outperforming existing kinase–substrate prediction methods (19, 20, 34, 35, 36).

To facilitate the use of this network as an alternative to literature-based pathways, we developed a web server that extracts context-specific signaling networks from global phosphoproteomics datasets and provides additional functional analyses. This tool significantly enhances and extends the functionalities of the original SELPHI web server (8), offering a more accurate network inference framework rather than relying on simple correlation-based kinase-substrate associations. The SELPHI_2.0_ web server is freely accessible at https://selphi2.com.

## Experimental Procedures

### Generation of Kinase-Substrate Probabilistic Network

Information on known kinase-substrate relationships and a comprehensive list of curated phosphopeptides were extracted from PhosphoSitePlus (37) (Downloaded on 03.09.2024). Functional scores and predictive features on phosphosites were downloaded from Ochoa and colleagues (38) (Supplemental Table 1). Proteomics data sets for co-regulation were downloaded from Mertins and colleagues (39) and Hijazi and colleagues (33). Expression data was downloaded from GTEx (40) (Downloaded on 03.09.2024) and the Human Protein Atlas version 23.0 (41) (Downloaded on 03.09.2024). Experimentally supported kinase–substrate relationships were downloaded from two recent publications (32, 33). The complete feature table for all kinase–substrate pairs tested can be found in Supplemental Table 2.

Predictions were made between 421 kinases, and 238,374 phosphosites (199,262 Ser/Thr & 39,112 Tyr) found on 17,469 proteins that were listed in PhosphositesPlus (37) and had a functional score (38) assigned to them.

Kinases annotated with tyrosine and serine/threonine in PhosphoSitePlus (37), as well as those identified as dual-specificity kinases in their NCBI gene summaries, were classified as prior knowledge dual-specificity kinases, and for those 33 kinases, predictions were made on all 238,374 phosphosites.

As a positive training set, we used 14,542 kinase-phosphosite relationships extracted from PhosphositePlus (37) (Downloaded on 03.09.2024). As there are no databases with information on known negative kinase-substrate pairs and given the fact that biological networks tend to be sparse, random samples of kinase-substrate relationships 10, 20, and 50 times as large as the positive set were used as a negative training set. There were no differences between the results, we therefore present those where we used a negative set 50 times as large as the positive set.

We generated or acquired 48 features (see below and Supplemental Table 1) based on various metrics that could influence kinase-substrate interactions. These included co-regulation in high-throughput datasets and phosphopeptide matching to kinase specificity profiles, captured by PSSMs. To identify the most informative predictors, we evaluated different feature combinations using 100 training/testing datasets.

For feature selection, we constructed 100 training datasets by combining our positive set with a negative set, which consisted of randomly generated kinase-substrate relationships 10 times larger than the positive set. We then trained random forest classifiers (42) using *scikit-learn* (43) to predict kinase-substrate interactions.

The random forest algorithm includes several tunable parameters. We set bootstrap sampling to TRUE and optimized three key parameters via grid search:

• max_depth (maximum tree depth): [10, 20, 50, 70, 80, 100]
• min_samples_split (minimum samples required to split a node): [8, 10, 12]
• n_estimators (number of trees in the forest): [150, 500, 1000, 1500]

All parameter combinations were tested using GridSearchCV(), as implemented in *scikit-learn*.

To rank feature importance, we applied recursive feature elimination with cross-validation (RFE-CV) using *scikit-learn* (43). We used ten-fold cross-validation to evaluate each model’s performance, selecting the best feature set based on the area under the ROC curve (AUC-ROC).

Finally, we selected 45 features for the final model using a majority voting approach, retaining those that appeared in >50% of the top-performing models (Supplemental Table 1).

For training the final model we used the random forest algorithm (42) as implemented in the *scikit-learn* python library (43). The model was trained using the positive set and a random sample of negative relationships, repeating the process 100 times. In each run, grid search was used to optimize parameters as described above. Ten-fold cross-validation was performed to validate each model. To ensure a balanced training and test set, we applied a stratified K-fold split, as implemented in *scikit-learn* (43), maintaining the same ratio of positive and negative labels across all splits. The final probability of each kinase–substrate interaction was assigned based on the average probability across all model outputs.

To assess the predictive power of our model on previously uncharacterized kinase-substrate interactions, we compared our predictions to experimentally supported interactions from two recent studies (32, 33). We then evaluated whether these experimentally supported relationships were assigned higher probabilities by our method. To quantify predictive performance, we calculated the area under the ROC curve (AUC-ROC) using the *ROCR* package (44).

### Comparison with Other Methods

To compare SELPHI_2.0_ with other state-of-the-art peer-reviewed methods, we assessed how well our method predicted known annotated kinase-substrate relationships (see positive/negative training sets described above) and novel kinase–substrate relationships supported by experimental procedures. These were derived from the recent works of Sugiyama and colleagues, and Hijazi and colleagues (32, 33), excluding kinase-substrate phosphorylation relationships found in PhosphoSitePlus, to limit literature-based biases.

We compared our method with six other established and most commonly used methods: PhosphoPICK (20), GPS v.5.0 (36), GPS v.6.0 (24), KinomeXplorer (19), NetPhos v.3.1 (34) and LinkPhinder (35). PhosphoPICK and NetPhos v3.1 are available as web servers and sequences of substrate proteins were uploaded onto the servers. For both PhosphoPICK and NetPhos v3.1, we selected no significance threshold to include all predictions. For NetPhos v3.1 we selected all available residues: Serine, threonine, and tyrosine. GPS was downloaded from (http://gps.biocuckoo.cn/download.php), and batch kinase prediction was run on Ubuntu 22.04. The confidence threshold was set to “All” to include all predictions. The KinomeXplorer prediction software was downloaded and predictions were made for the phosphosites included in our network. All 11,581,940 LinkPhinder predictions were downloaded from (https://linkphinder.insight-centre.org/download).

These methods were individually compared with SELPHI_2.0_ by assessing their ability to capture known kinase substrate relationships from PhosphoSitePlus. As the published methods were able to make predictions for different subsets of kinases, we restricted the comparisons to the intersection of the available predictions (Supplemental Table 3). For each method under comparison, we generated 100 validation sets made from the positive set and a negative set selected from random kinase substrate relationships that were present both in SELPHI_2.0_ and the method that was under comparison. The negative set was 10 times larger than the positive set.

The ability of SELPHI_2.0_ to discriminate between known interactions and interactions with unknown status was then compared to each of the other methods by calculating the average AUC achieved by SELPHI_2.0_ and the method under comparison for the 100 different validation sets. Only kinase substrate relationships that were shared by both methods were included in each run.

To evaluate the ability of our method to capture novel kinase–substrate interactions, we used an independent test set of experimentally predicted kinase-substrate relationships from Sugiyama *et al*. (32), as described earlier. We compared the performance of SELPHI_2.0_ against each method individually, using the area under the ROC curve (AUC-ROC) as the performance metric. In each pairwise comparison, the performance was evaluated on the intersection of the predictions available for both methods. AUC scores were calculated using the *ROCR* package (44).

### Evaluation of Model Fit to Phosphoproteomic Data

To capture context-specific signaling networks, we constructed a reference SELPHI_2.0_ signaling network by linking the kinase-substrate predictions made in this work to a backbone of a probabilistic kinase-kinase regulatory network that we had previously published (28). A probability cutoff of 0.5 was applied to both networks to select higher confidence edges.

High-throughput mass spectrometry-based phosphoproteomic data that had been compiled and analyzed by Ochoa and colleagues with 436 conditions (45) were fitted to our network by means of the PCSF algorithm (46). We tried all combinations of the of the following PCSF parameters: For the tree parameter, b, we tried fitting anything from 1 to 10 trees to the data, for parameter w or the node tuning we tried the following values: 0.25, 0.5, 0.75, 1.0, 1.25, 1.5 and for the edge tuning, *μ*, we tried 0.000005, 0.00005, 0.0005, 0.005, 0.05. Edge probabilities subtracted from one were used as edge costs. These parameter combinations generated 436 subnetworks that gave the highest F1 score. To evaluate the performance of the fitting, the F1 score of kinase–substrate phosphosite relationships retained after the fitting was compared with those that were used in the input.

### Counting Number of Citations Related to Proteins

To count the number of citations related to kinases and their substrates we used the Entrez.elink() function from the Biopython module (47). We searched for related articles in the Pubmed database. Linked publications were retrieved from NCBI Entrez Gene (48) database and publications that mention more than 10 kinases were filtered out.

### Systems-Level Analysis of SELPHI_2.0_ network

We computed the similarity between kinases based on the predicted substrates, namely, considering all kinase-phosphosite associations having a SELPHI_2.0_ score greater than 0.5. Distances between each pair of kinases were quantified as 1 - J(A,B), where J(A,B) is the Jaccard index of the sets of substrates of the two kinases, therefore obtaining a distance matrix. Hierarchical clustering between kinases was performed using the unweighted pair group method with arithmetic mean (UPGMA (49)) on the distance matrix using the UPGMA implementation in SciPy 1.15.2 (50). Radial dendrograms for visualizing hierarchical kinase similarity were generated with a custom Python module. The same methodology was also used to evaluate the kinase hierarchical similarity based on the Gene Ontology terms enrichment of their predicted substrates, with the only difference being J(A,B) representing the Jaccard index of the sets of terms significantly associated, according to the tests described above, to two kinases A and B. For substrate enrichment analysis, we used the enrichGO function from the *clusterProfiler* R package to analyze all substrates with phosphosites with a SELPHI_2.0_ score >0.5 for each kinase individually against Gene Ontology biological processes. Subsequently, we filtered the results for leaf nodes retrieved from the *topGO* R package using all terms without any children (GOBPCHILDREN). The enrichments performed during the analysis on the understudied kinases was generated using Fisher’s Exact test using Reactome as the gene sets and an adjusted *p*-value cutoff <0.05. Reactome was chosen as the background database for this analysis to prioritise kinase similarities related to specific pathways for interpretability.

We mapped kinases to their respective families using the CORAL kinase group classification (51), and for kinases not present in the map, we assigned them to the family of their respective paralogs. 12 kinases could not be assigned to a kinase family. Subsequently, we visualized the number of known phosphosites and predicted SELPHI_2.0_ phosphosites using the CORAL website (http://phanstiel-lab.med.unc.edu/CORAL/).

A list of dark kinases was downloaded from the Dark Kinome Knowledgebase (https://darkkinome.org/) (52). Additionally, we created a second dark kinase annotation by taking the bottom 20% kinases with the fewest citations from the section above.

### Implementation of SELPHI_2.0_ Web Server

The SELPHI_2.0_ web server has been implemented using the *shiny* (53) R package. Enrichment analyses on the server are performed using the *enrichR* (54) and the *clusterProfiler* (55) enrichment package, and users will be able to select gene sets for enrichment from various databases: Reactome (6), KEGG (5), GO (56), DISEASES (57), BioPlanet (58) and PTMSigDB (59) (for phosphosite-level enrichment). For phosphopeptide clustering, the two clustering algorithms available are Gaussian mixture models as implemented by *mclust* (60) and k-means clustering (61). If k-means clustering is selected, silhouette index (62) is used to calculate the optimal number of clusters while *mclust* uses the Bayesian information criterion to select the best fitting model (63). The output is presented as a dot plot where the color indicates the sum of the log_2_ ratios of the phosphosites involved in the enrichment term and size the odd ratio of the enrichment term.

For the calculation of kinase activities, the web server uses the 95th percentile of kinase-substrate probabilities to select high-confidence kinase substrates. We then use the FGSEA-multilevel algorithm as implemented in the *fgsea* R-package (64) to assess if the predicted substrates are overrepresented at the upper or lower end of the log_2_ ratio distribution for a given condition. The normalized enrichment scores of kinases with an adjusted *p*-value ≤0.05 are plotted to quantify their activities. For dual-specificity kinases, the enrichment analysis is run separately on Ser/Thr and Tyr residues. For comparison, kinase activities using the PhosphositePlus (37) and the Sugiyama (32) kinase–substrate data set are also calculated.

## Results

### Generation of a Probabilistic Human Kinase-Substrate Regulatory Network

**Fig. 1:**
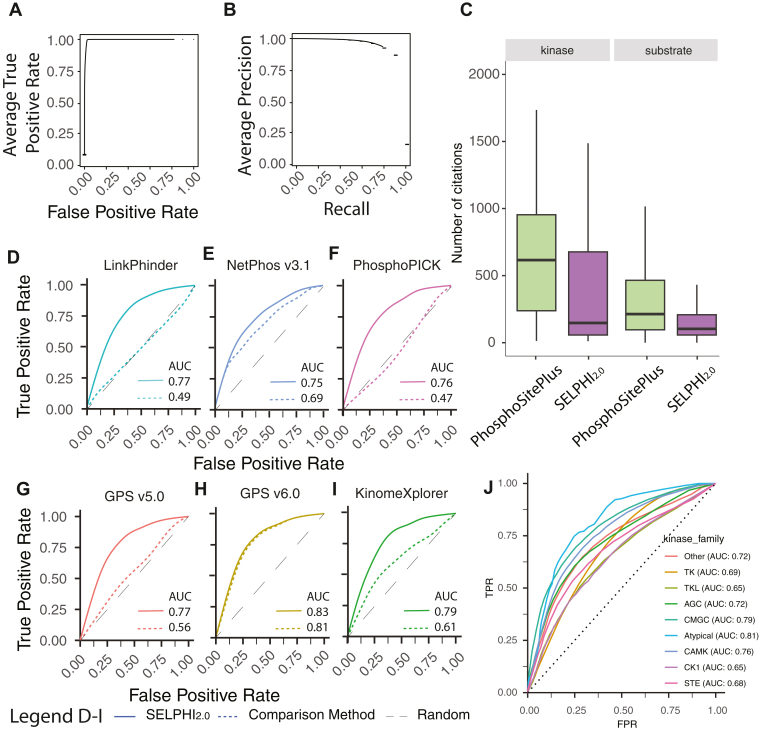
**Performance of SELPHI_2.0_**. *A*, predictive power of the SELPHI_2.0_ model. Average ROC curve (AUC 0.97) after 100 runs of cross-validation with random sets of negative examples and a positive set from PhosphositePlus. *B*, average precision-recall curve for the same cross-validations. *C*, SELPHI_2.0_ makes high confidence predictions for less cited kinases and less cited substrates compared to kinase-substrate pairs found in PhosphositePlus. *D–I*, comparison between SELPHI_2.0_ (solid lines) and other state-of-the-art kinase-substrate prediction methods (dashed) with respect to the ability of each method to discern between experimentally supported kinase-substrate relationships and the rest of the relationships unsupported by the experiments. *J*, ROC curves for the performance of SELPHI_2.0_ on experimentally supported kinase-substrate associations not present in the literature, per individual kinase family.

To create a probabilistic network of kinase-substrate relationships, we used the random forest algorithm to combine various predictive variables ranging from co-phosphorylation and co-expression in large datasets, to kinase–substrate sequence specificities and features related to the functionality of the phosphosites (Experimental Procedures; Supplemental Tables 2 & 4). After running the prediction algorithm 100 times, we achieved an average AUC of 0.97 (Fig. 1, *A* and *B*). Of the 73,207,860 predictions made, 721,707 edges were classified as likely regulatory (probability >0.5). Of those, around 1.7% were found in PhosphoSitePlus (37) while more than 90% of the known interactions found in our network were assigned a high (>0.5) probability.

Importantly, our network provides predictions covering the ‘dark’ or less well studied human signaling network. Specifically, kinase–substrate relationships found in PhosphositePlus have a median number of 685 citations per phosphorylating kinase and 232 per substrate respectively. In contrast, the median number of citations for our regulatory predictions (>0.5 probability) is 194 for the kinases and 96 for the substrates, meaning that we provide proportionally more predictions between kinases and substrates with significantly lower number of citations per protein on average (Kinases: W = 3.37 × 10^9^, *p* < 2.2 × 10^-16^, one-sided Wilcoxon test. Substrate proteins: W = 3.34 × 10^9^, *p* < 2.2 × 10^-16^, one-sided Wilcoxon test) (Fig. 1*C*). These predictions include substrates that have not been mentioned in the literature before and have no known upstream kinase, highlighting the value of this network as a reference to explore the less studied part of the phospho-signaling network.

### SELPHI_2.0_ Outperforms the State-of-the-Art Methods for Kinase–Substrate Prediction

To evaluate our method’s ability to predict novel kinase-substrate interactions, we first used cross-validation on the training set (Experimental Procedures). In comparison to six state-of-the-art kinase-substrate prediction methods (PhosphoPICK (20), GPS v.5.0 (36) & v.6.0 (24), KinomeXplorer (19), NetPhos v.3.1 (34) and LinkPhinder (35)), SELPHI_2.0_ performs generally better on identifying known kinase-substrate interactions (Supplemental Fig. S1), while making predictions for more kinases.

We next assessed how well it distinguished between experimentally predicted kinase-substrate relationships that had not been previously reported in the literature and other unsupported (putatively negative) relationships. For this, we used interactions identified in two recent studies (32, 33) after removing those among them that already exist in PhosphoSitePlus. In brief, the one study uses an LC-MS/MS-based *in vitro* kinase assay to identify substrates for 385 kinases (32, 33), while the other treats cell lines with kinase inhibitors and uses a new statistic to infer kinase-substrate relationships based on the resulting changes in phosphorylation (32, 33).

In this task, we found that our network performs better than the others (Fig. 1, *D*–*I*) with GPSv.6.0 performing the closest with AUC = 0.81 compared to our method’s 0.83, albeit making predictions for 349 kinases instead of SELPHI_2.0_’s 421. NetPhos v3.1 achieved an AUC of 0.69 compared to SELPHI_2.0_’s AUC of 0.75, but made predictions for only 17 kinases. KinomeXplorer (19) achieved an AUC of 0.61 compared to SELPHI_2.0_’s AUC of 0.81 for the same set. Other methods performed close to random.

When looking at the performance per kinase family the Atypical and CMGC kinases had the best performance (AUCs 0.81 and 0.79 respectively), whereas the worst performance was achieved by the CK1 and TKL families (AUCs 0.65 for both), likely due to the low number of experimentally identified true positive substrates for these groups (Fig. 1*J*).

Within our positive kinase-substrate training dataset, we identified 33 kinases that appeared to have dual specificity as they had annotations for both tyrosine and serine/threonine phosphosites. However, when looking at the distribution of our predictions we found that only five of these are likely to be indeed dual specificity, namely MAP2K6, MAP2K3, MAP2K4, MAP2K7 and MAP2K2 and potentially also STK16, but with less confidence as we only had three predictions for it (Supplemental Fig. S2). The rest of them should be revisited as they may suffer from erroneous annotations in the reference database. For example, looking at the serine phosphosites assigned to EGFR, which is a well-studied receptor tyrosine kinase, we find that the associated publications were providing indirect evidence for phosphorylation of these sites downstream of EGFR activation, but not direct phosphorylation by EGFR (65, 66, 67).

### SELPHI_2.0_ can be Used to Identify Novel High-Confidence Kinase–Substrate Relationships

**Fig. 2:**
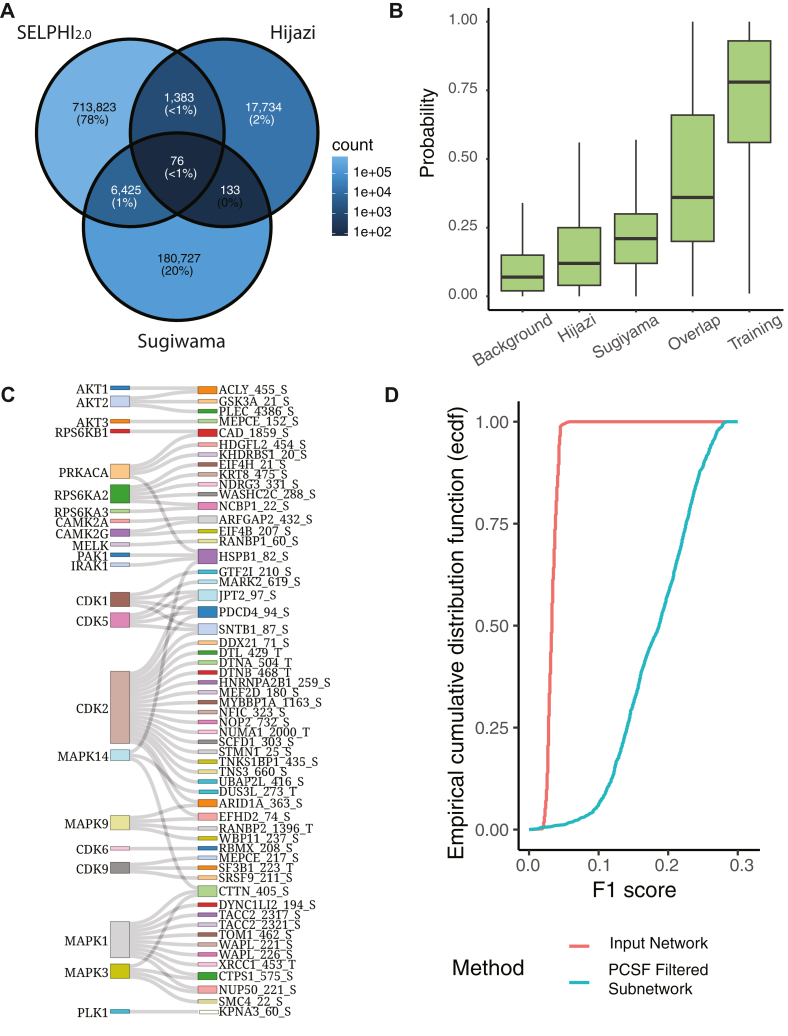
**Identification of high confidence kinase-substrate interactions**. *A*, Venn diagram showing the overlap between SELPHI_2.0_’s predictions and two studies that generated experimentally supported kinase-substrate relationships. The color reflects the number of relationships represented in each circle: darker indicates less relationships. *B*, experimentally validated kinase-substrate relationships were assigned a higher probability compared to the background with kinase-substrate predictions made by both methods being assigned the highest probabilities of all sets. *C*, Sankey plot of the 76 high confidence kinase-substrate relationships that were supported by both experimental studies and our predictions. *D*, fitting the kinase-substrate predictions to experimental data selects for known kinase-substrate relationships with median F1 score of 0.031 for the unfitted sub network compared to 0.19 for the fitted input.

Generally, we found that the overlap between the two experimental sets and the SELPHI_2.0_ predictions was relatively low (Fig. 2*A*). Due to the complementary characteristics between the two experimental methods, we reasoned that kinase-substrate relationships identified by both studies should have higher levels of confidence. Our method assigned significantly higher probabilities to experimentally supported edges compared to the rest of the network (Fig. 2*B*). Furthermore, we found that kinase-substrate relationships supported by edges found in both data sets had an even higher probability assigned to them (Fig. 2*B*) compared to edges supported by either.

By overlapping the kinase-substrate relationships supported by both experimental studies and SELPHI_2.0,_ we identified 76 novel high-confidence interactions. Thirty (30) among these involved novel substrate associations with CDK2 and MAPK1 (Fig. 2*C*; Supplemental Fig. S3*A*), and the substrate involved in the most predictions (4) was the Heat shock protein - b1 (HSPB1), a molecular chaperone with roles in stress resistance and actin organisation (68) (Fig. 2*C*; Supplemental Fig. S3*B*). Several of these have orthogonal support - for example the CDK2-JPT2 S97 relationship has been observed on an additional *in vitro* study to find CDK2 substrates (69), whereas the AKT-ACLY S455 interaction is already annotated as putative in PhosphoSitePlus (37, 70). Thus, the 76 high-confidence kinase-substrate associations are strong candidates for downstream experimental validation.

### Extraction of Dataset-Specific Networks From Our Reference Network Selects for Known Interactions

Our predicted kinase–substrate interaction network assigns probabilities to kinase phosphorylation events based on all features incorporated in the model. While the model performs well and the resulting network is enriched in known interactions, the large number of predicted interactions means that many false positives are likely present. We hypothesized that using our network as a reference to fit phosphoproteomics datasets would further enrich true interactions, helping to filter out potential false positives and enabling the unbiased extraction of dataset-specific signaling networks.

To test this, we fitted our network to a set of mass spectrometry-based global phosphoproteomic data sets generated under different conditions. We used a compilation of recently reanalyzed mass spectrometry data sets (45). To fit our predictions to high-throughput data, the predicted kinase-substrate edges were combined with a kinase-kinase regulatory network that we had generated previously (28), forming a network of kinase-kinase regulatory relationships with phosphosites as nodes without outgoing edges (Experimental Procedures). To select high-confidence edges, we used an edge probability of 0.5 as a threshold for both the kinase–kinase regulatory network and the kinase-substrate predictions.

To fit the combined network to the high-throughput data sets, we used the Prize collecting Steiner’s forest algorithm (46). We found that by optimizing the edge cost against the node prizes we were able to enrich previously known kinase-substrate relationships. The F1 scores of the fitted subnetworks (n = 415) were 0.19 compared to the unpruned input with a F1 score of 0.031. The improvement in precision was even greater with the mean precision of the pruned subnetworks being 0.22 while the precision was 0.016 for the unpruned input networks. Both comparison yielded a significant difference (F1 score: W = 2.14 × 10^6^, *p* < 2.2 × 10^-16^, precision: W = 2.14 × 10^6^, *p* < 2.2 × 10^-16^), indicating this combination of kinase-kinase regulatory network and kinase-substrate predictions can be used to extract high probability context-specific subnetworks (Fig. 2*D*).

### The SELPHI_2.0_ Resource can Help Annotate Understudied Kinases

**Fig. 3:**
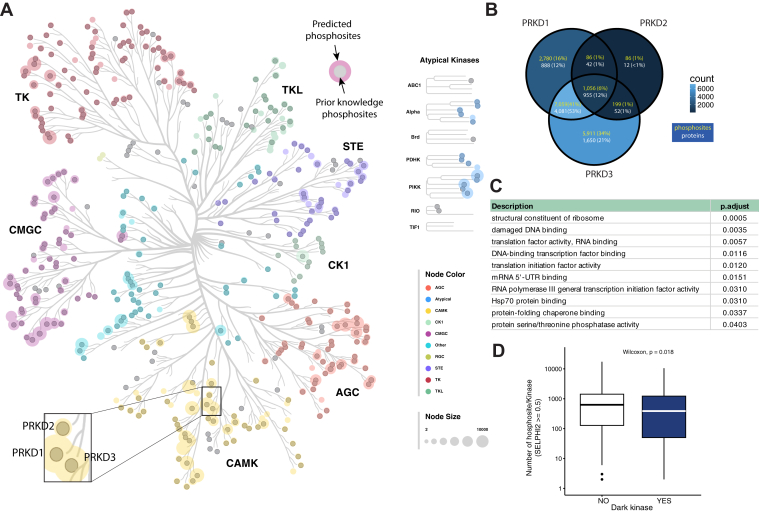
**Overview of SELPHI_2.0_’s predictions across the kinome tree**. *A*, distribution of our predictions and comparison with existing known relationships across the kinome tree. *B*, example of predictions numbers and overlaps for three paralog kinases. *C*, functional enrichment analysis of the unique substrates of PRKD3 indicate a function in translation related processes and damaged DNA binding. *D*, SELPHI_2.0_ makes fewer predictions for the kinases annotated as “dark” in the Dark Kinome Knowledgebase.

We next explored our probabilistic kinase-substrate map for potential systems-level observations. We first explored the distribution of the numbers of predicted substrates across kinases. Among kinase families we found that the median number of relationships predicted was roughly similar with the CMGC family having the most associations (median >1000) and the tyrosine kinases (TK, TKL), having slightly less than most of the serine/threonine kinase families (Fig. 3*A*, Supplementary Fig. S4). The CK1 family was the exception for which we found ∼1-0.5 orders of magnitude less regulatory relationships than the rest of the serine/threonine kinase families, likely to the small number of kinases represented and the fact that three of these were “dark” kinases (Fig. 3*A*, Supplementary Fig. S4).

The kinases with the most associations had more than 10,000 predicted associations with CHEK1, CDK1, RPS6KA4, RPS6KA2, MAPK1 and PRKD3 being the most promiscuous among the kinases in our results (Supplemental Table 3, Supplemental Fig. S5*A*). PRKD3 is not annotated in the Dark Kinase Knowledgebase, however presently has very few functional annotations (assigned due to similarity to its paralogs), only five known substrates and less than 45 associated citations. It is one of three paralog kinases (PRKD1,2 and 3) that are involved in Diacylglycerol (DAG) signaling downstream of the PKC kinase. For the three paralogs 1056 phosphosites were common targets, 7059 were common between PRKD3 and PRKD1, 199 between PRKD1 and PRKD2 whereas 5911 were unique to PRKD3 (Fig. 3*B*). Reassuringly, PRKD1 and PRKD3 had more substrates in common than PRKD2, which is more evolutionarily distant (Fig. 3*A*). An enrichment analysis of the unique PRKD3 phosphoproteins, showed enrichment in translation-related processes and damaged DNA binding (Fig. 3*C*), which suggests a distinct function for this kinase in addition to the ones it shares with its paralog proteins PRKD1 and 2, downstream of PKC signalling and cytoskeleton regulation.

The kinases with the least number of predicted target phosphosites were TNNI3K, NEK10, MAPK4, CSNK1G3, and STK16 with three or fewer substrates identified (Supplemental Fig. S5*B*). No substrates were predicted for ACVR1C, DCLK3, DSTYK, STRADB. Reassuringly, STARDB is annotated as a pseudokinase (71). Several of the kinases with very few or no predicted substrates are included in the Dark Kinase Knowledgebase (52), which includes 160 kinases that are considered to be very understudied.

Looking at our predicted network, we found that we have made a median of 391 predictions per dark kinase, in contrast to 629.5 predictions for the better studied kinases suggesting that dark kinases may be more specific in their phosphorylations than well-studied ones (Wilcoxon *p* value = 0.018; Fig. 3*D*). However, when we checked whether the substrates of the dark kinases are annotated with generally less unique GO terms (leaf terms) than those of the rest of the kinases, which we used as a proxy to test whether they indeed have more specific functions, we only found this to be marginally true (5% difference, *p*-value <2.2e-16). This suggests that although SELPHI_2.0_ is more unbiased and accurate than other available tools, there may still be some inherent bias leading to generally more predictions for the well-studied kinases. Nonetheless, SELPHI_2.0_ provides predictions for 51.7% of known phosphosites as opposed to 4.2% that are currently annotated and 8.9% and 2.6% provided in the Sugiyama *et al* (32), and Hijazi *et al* (33) studies respectively.

**Fig. 4:**
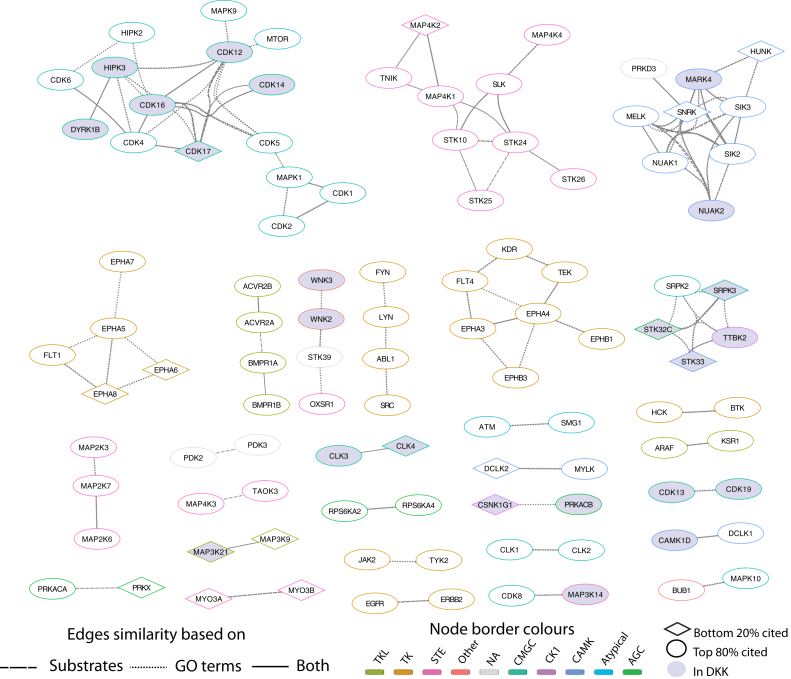
**Networks of kinases with the highest overlap of predicted substrates**. Only kinase edges that represent a Jaccard index of >0.5 have been included. Node border color indicates the kinase family. Dotted edges represent similarity of GO terms of the substrates, dashed line represents similarity of substrates themselves. Diamonds indicate the 20% least cited kinases and light violet indicates kinases included in the Dark Kinome Knowledgebase (DKK).

Finally, we explored whether we can propose functions for the understudied or ‘dark’ kinases based on the functions of their predicted substrates. Clustering all kinases according to the overlap of their predicted substrates showed good agreement at the level of the kinase family (e.g. TK family kinases were mostly clustered together, and the same goes for CMGC kinases with other families (e.g. STE) having a more diverse range of substrates (Supplementary Fig. S6). When looking only at groups of kinases that had a high overlap in their predicted substrates (Jaccard index >0.5) we found 108 kinases forming 10 clusters of three or more kinases and several pairs (Fig. 4) and explored whether these could be useful for annotating any of the 23 kinases that were found in the clusters and were present in the Dark Kinase Knowledgebase, or any of the 16 kinases in the network that were on the bottom 20% of the rank based on kinase citations. For example, one cluster comprised OXSR1, STK39, WNK2, and WNK3 (Fig. 4). Despite WNK2 and WNK3 being in the Dark Kinase Knowledgebase, there are predicted annotations by similarity with respect to their function in activating STK39 and OXSR1 for the regulation of ion cotransporters in the Uniprot database (72). Indeed, the main Reactome pathway that is enriched in common across the substrates of this cluster is “Cation-coupled Chloride Cotransporters”. Similarly, a cluster that includes EPHA7, EPHA5, and FLT1, which have common functionality in neuronal development and axon guidance, also includes EPHA8 and EPHA6, which are poorly cited kinases with limited predicted annotations(Fig. 4). Using our resource, we propose that EPHA8 and EPHA6 may also contribute to these functions. The heavily understudied SRPK3, STK32 C, STK33, and TTBK2 kinases are also clustering with SRPK2(Fig. 4), a kinase that regulates splicing, and their substrates are commonly enriched in functions related to “mRNA Splicing”, “Processing Of Capped Intron-Containing Pre-mRNA,” and other similar terms. Overall, this kind of use of our resource can generate hypotheses regarding the function of both understudied kinases and non-annotated substrates.

### SELPHI_2.0_ Web Server

We have created a publicly available web server, SELPHI_2.0_, that allows less biased analyses and interpretation of global phosphoproteomics datasets by considering context-specific and understudied signaling processes. The basis of this web server is the data-driven network of kinase substrate predictions described above. SELPHI_2.0_ improves on an earlier resource SELPHI (Systematic Extraction of Linked Phospho-Interactions) (8), which calculated kinase-substrate associations based on correlations and filtering based on prior knowledge.

**Fig. 5:**
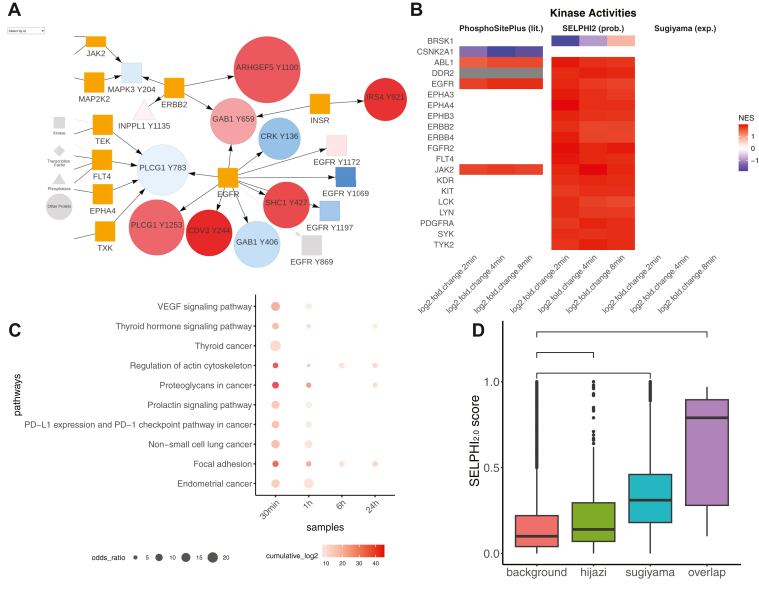
**Examples of the SELPHI_2.0_ web server outputs**. *A*, example of fitting the user’s data to the SELPHI_2.0_ network. Red to blue indicates the log-fold change for the specific node, gray nodes are those that are present in the networks generated by other samples in the same dataset but have no data available in the current condition and the shapes of the nodes correspond to different types of proteins (kinase, transcription factors, phosphatases, other proteins). *B*, kinase activities across the different conditions or time points calculated based on the Sugiwama *et al* dataset (experimental), PhosphoSitePlus prior knowledge associations (literature) and SELPHI_2.0_ (probabilistic). *C*, example of functional enrichment analysis output from the full dataset. *D*, the user can assess if experimentally predicted edges from independent studies corroborate the prediction made by SELPHI_2.0_. Furthermore, the user can download a list of these experimentally corroborated edges. Köksal, 2018, the first example in the web server, was used for (*A*), (*B*), and (*D*) and Barrio-Hernandez, 2020, the second example in the web server, was used for (*C*).

Users can use SELPHI_2.0_ to retrieve the top kinase-substrate predictions for the phosphosite included in their data. They can also identify the active signalling sub-networks in their data by mapping it to our SELPHI_2.0_ reference kinase-substrate network (Experimental Procedures; Fig. 5*A*). Furthermore, they can extract condition specific kinase activity profiles based on SELPHI_2.0_’s predictions, prior knowledge or the in vitro-supported kinase substrate relationships (32) (Fig. 5*B*). Standard functional enrichment analyses are also supported, using prior knowledge pathways (Reactome (6), KEGG (5), GO (56), DISEASES (57), BioPlanet (58); Fig. 5*C*), or at the phosphosite level (PTMSigDB (59)) either on their up/downregulated phosphosites or on clusters of phosphosites identified through the server. Finally, the server also provides higher confidence relationships by overlapping our predictions with the two experimentally derived data sets published previously (32, 33). This way the user can prioritize edges that have been corroborated by independent experimental results as well as large scale omics datasets (Fig. 5*D*).

## Discussion

Kinase-substrate networks form the backbone of cell signaling responses and are critical for cell function in health and disease (73). However, the vast number of potential kinase-substrate interactions, most of which remain uncharacterized, necessitates computational approaches for accurate annotation and prioritization. Here, we present SELPHI_2.0_, a machine learning-based probabilistic kinase-substrate network at the phosphosite level, covering 421 kinases and 238,374 phosphosites.

SELPHI_2.0_ outperforms five state-of-the-art methods (19, 20, 34, 35, 36) on benchmark datasets and, importantly, on entirely novel experimentally supported interactions (32, 33). Despite making predictions for more kinases than other methods, SELPHI_2.0_ maintains high accuracy, making it a strong starting point for prioritizing novel kinase-substrate relationships. GPS v.6.0 (24) was an exception, performing very similarly to SELPHI_2.0_ but being limited to 349 kinases. When overlaying our predictions with the two experimentally supported datasets, the resulting 76 high-confidence kinase–substrate relationships are supported by both *in vitro* and cell line-based experimental data, and our data-driven machine learning approach, giving them particularly high confidence.

Unlike many existing models, SELPHI_2.0_ leverages high-throughput, unbiased functional genomics datasets rather than relying solely on literature-based knowledge, helping to reduce bias in cell signaling research. Our network significantly expands coverage of the kinase signaling landscape while improving prediction accuracy. The median citation count for kinases and substrates in SELPHI_2.0_ is 3.5 and 2.4 times lower than those in PhosphoSitePlus, highlighting its potential to explore underrepresented areas of cell signaling. Indeed, in our study, we showcase how our resource could be used to generate hypotheses about the function of understudied kinases. Given the importance of prior networks in signaling inference, SELPHI_2.0_ provides a more comprehensive and accurate foundation for studying understudied kinases in the context of current knowledge.

SELPHI_2.0_ integrates the latest kinase specificity maps (PSSMs) (26, 27) with functional information from high-throughput datasets, enhancing kinase activity estimation from phosphoproteomics data. Tools such as PhosX (74), which use kinase specificity scores rather than known substrates, could benefit from SELPHI_2.0_’s scores, incorporating additional metrics like co-expression to refine kinase activity predictions.

To make SELPHI_2.0_ widely accessible, we developed a web server that facilitates global phosphoproteomics data analysis for downstream experimental design. Phosphoproteomics datasets capture context-specific signaling networks, but their inherent noisiness and sparsity make unbiased network extraction challenging (4). SELPHI_2.0_ improves upon the original SELPHI web server, which relied on correlation-based kinase-substrate associations filtered by prior knowledge, inadvertently reinforcing literature bias and producing noisy predictions for understudied kinases.

With SELPHI_2.0_, users can extract dataset-specific networks from our probabilistic reference network using PCSF. Given that signaling responses vary by cell type, condition, and tissue state, and that current pathway databases poorly explain phosphoproteomics data, context-specific network inference is essential. Future improvements could incorporate more sophisticated statistical approaches, such as MRA (75) or Integer Linear Programming methods (76).

False positives are an inherent challenge in kinase-substrate predictions, partly due to the large portion of the signaling network that remains uncharacterized. While SELPHI_2.0_ is not immune to this issue, its strong performance compared to existing methods suggests it provides a robust framework for hypothesis prioritization. As higher quality phosphoproteomics data sets become available, SELPHI_2.0_ can be further refined.

In conclusion, SELPHI_2.0_ facilitates the discovery of novel kinase-substrate interactions, aiding both small-scale signaling studies and large-scale network inference. Its web server makes these insights accessible to non-bioinformatics experts, providing a powerful tool for unbiased and context-specific phosphoproteomics analysis. By enabling data-driven experimental design, SELPHI_2.0_ has the potential to advance our understanding of signal transduction across diverse cellular conditions and contexts.

## Data Availability

The SELPHI_2.0_ web server is freely available under: https://selphi2.com and all code for reproducing this project can be found at https://gitlab.ebi.ac.uk/petsalakilab/selphi_2.0 and https://github.com/alussana/selphi2.0-pssms. The code for generating the kinase radial trees can be found here: https://github.com/alussana/radialtree. A Docker image of the web server can be found at: https://gitlab.ebi.ac.uk/petsalakilab/selphi_2. The feature table, positive training set, full prediction results and citation matrix can be found in Zenodo: https://doi.org/10.5281/zenodo.13821320.

## Supplemental Data

This article contains supplemental data (77, 78, 79, 80, 81, 82, 83, 84, 85, 86, 87, 88, 89, 90).

## Conflict of Interest

The authors declare that they have no conflicts of interest with the contents of this article.

## References

[1] Pawson T., Saxton T.M. (1999). Signaling networks—do all roads lead to the same genes?. Cell.

[2] Hunter T. (1995). Protein kinases and phosphatases: the yin and yang of protein phosphorylation and signaling. Cell.

[3] Bhullar K.S., Lagarón N.O., McGowan E.M., Parmar I., Jha A., Hubbard B.P. (2018). Kinase-targeted cancer therapies: progress, challenges and future directions. Mol. Cancer..

[4] Needham E.J., Parker B.L., Burykin T., James D.E., Humphrey S.J. (2019). Illuminating the dark phosphoproteome. Sci. Signal..

[5] Kanehisa M., Furumichi M., Tanabe M., Sato Y., Morishima K. (2017). KEGG: new perspectives on genomes, pathways, diseases and drugs. Nucleic Acids Res..

[6] Jassal B., Matthews L., Viteri G., Gong C., Lorente P., Fabregat A. (2020). The reactome pathway knowledgebase. Nucleic Acids Res..

[7] Türei D., Korcsmáros T., Saez-Rodriguez J. (2016). OmniPath: guidelines and gateway for literature-curated signaling pathway resources. Nat. Methods..

[8] Petsalaki E., Helbig A.O., Gopal A., Pasculescu A., Roth F.P., Pawson T. (2015). SELPHI: correlation-based identification of kinase-associated networks from global phospho-proteomics data sets. Nucleic Acids Res..

[9] Hill S.M., Heiser L.M., Cokelaer T., Unger M., Nesser N.K., Carlin D.E. (2016). Inferring causal molecular networks: empirical assessment through a community-based effort. Nat. Methods..

[10] Carlin D.E., Paull E.O., Graim K., Wong C.K., Bivol A., Ryabinin P. (2017). Prophetic Granger Causality to infer gene regulatory networks. PLoS One.

[11] Rodchenkov I., Babur O., Luna A., Aksoy B.A., Wong J.V., Fong D. (2020). Pathway Commons 2019 Update: integration, analysis and exploration of pathway data. Nucleic Acids Res..

[12] Zhou F., Xue Y., Yao X., Xu Y. (2006). A general user interface for prediction servers of proteins’ post-translational modification sites. Nat. Protoc..

[13] Neuberger G., Schneider G., Eisenhaber F. (2007). pkaPS: prediction of protein kinase A phosphorylation sites with the simplified kinase-substrate binding model. Biol. Direct.

[14] Li T., Li F., Zhang X. (2008). Prediction of kinase-specific phosphorylation sites with sequence features by a log-odds ratio approach. Proteins. Struct. Funct. Bioinf..

[15] Obenauer J.C., Cantley L.C., Yaffe M.B. (2003). Scansite 2.0: proteome-wide prediction of cell signaling interactions using short sequence motifs. Nucleic Acids Res..

[16] Huang H.D., Lee T.Y., Tzeng S.W., Horng J.T. (2005). KinasePhos: a web tool for identifying protein kinase-specific phosphorylation sites. Nucleic Acids Res..

[17] Ayati M., Yilmaz S., Lopes F.B.T., Chance M., Koyuturk M. (2023). Prediction of kinase-substrate associations using the functional landscape of kinases and phosphorylation sites. Pac. Symp. Biocomput..

[18] Brinkworth R.I., Breinl R.A., Kobe B. (2003). Structural basis and prediction of substrate specificity in protein serine/threonine kinases. Proc. Natl. Acad. Sci. U. S. A..

[19] Horn H., Schoof E.M., Kim J., Robin X., Miller M.L., Diella F. (2014). KinomeXplorer: an integrated platform for kinome biology studies. Nat. Methods.

[20] Patrick R., Lê Cao K.A., Kobe B., Bodén M. (2015). PhosphoPICK: modelling cellular context to map kinase-substrate phosphorylation events. Bioinformatics.

[21] Ayati M., Wiredja D., Schlatzer D., Maxwell S., Li M., Koyutürk M. (2019). CoPhosK: a method for comprehensive kinase substrate annotation using co-phosphorylation analysis. Plos Comput. Biol..

[22] Kim J.H., Lee J., Oh B., Kimm K., Koh I. (2004). Prediction of phosphorylation sites using SVMs. Bioinformatics.

[23] Xue Y., Li A., Wang L., Feng H., Yao X. (2006). PPSP: prediction of PK-specific phosphorylation site with Bayesian decision theory. BMC Bioinformatics.

[24] Chen M., Zhang W., Gou Y., Xu D., Wei Y., Liu D. (2023). Gps 6.0: an updated server for prediction of kinase-specific phosphorylation sites in proteins. Nucleic Acids Res..

[25] Wang D., Zeng S., Xu C., Qiu W., Liang Y., Joshi T. (2017). MusiteDeep: a deep-learning framework for general and kinase-specific phosphorylation site prediction. Bioinformatics.

[26] Yaron-Barir T.M., Joughin B.A., Huntsman E.M., Kerelsky A., Cizin D.M., Cohen B.M. (2024). The intrinsic substrate specificity of the human tyrosine kinome. Nature.

[27] Johnson J.L., Yaron T.M., Huntsman E.M., Kerelsky A., Song J., Regev A. (2023). An atlas of substrate specificities for the human serine/threonine kinome. Nature.

[28] Invergo B.M., Petursson B., Akhtar N., Bradley D., Giudice G., Hijazi M. (2020). Prediction of signed protein kinase regulatory Circuits. Cell Syst.

[29] Piwnica-Worms H., Saunders K.B., Roberts T.M., Smith A.E., Cheng S.H. (1987). Tyrosine phosphorylation regulates the biochemical and biological properties of pp60c-src. Cell.

[30] Pawson T., Scott J.D. (1997). Signaling through scaffold, anchoring, and adaptor proteins. Science.

[31] Mugabo Y., Lim G.E. (2018). Scaffold proteins: from Coordinating signaling pathways to Metabolic regulation. Endocrinology.

[32] Sugiyama N., Imamura H., Ishihama Y. (2019). Large-scale discovery of substrates of the human kinome. Sci. Rep..

[33] Hijazi M., Smith R., Rajeeve V., Bessant C., Cutillas P.R. (2020). Reconstructing kinase network topologies from phosphoproteomics data reveals cancer-associated rewiring. Nat. Biotechnol..

[34] Blom N., Sicheritz-Pontén T., Gupta R., Gammeltoft S., Brunak S. (2004). Prediction of post-translational glycosylation and phosphorylation of proteins from the amino acid sequence. Proteomics.

[35] Nováček V., McGauran G., Matallanas D., Vallejo Blanco A., Conca P., Muñoz E. (2020). Accurate prediction of kinase-substrate networks using knowledge graphs. Plos Comput. Biol..

[36] Wang C., Xu H., Lin S., Deng W., Zhou J., Zhang Y. (2020). Gps 5.0: an update on the prediction of kinase-specific phosphorylation sites in proteins. Genomics. Proteomics. Bioinformatics..

[37] Hornbeck P.V., Zhang B., Murray B., Kornhauser J.M., Latham V., Skrzypek E. (2015). PhosphoSitePlus, 2014: mutations, PTMs and recalibrations. Nucleic Acids Res..

[38] Ochoa D., Jarnuczak A.F., Viéitez C., Gehre M., Soucheray M., Mateus A. (2020). The functional landscape of the human phosphoproteome. Nat. Biotechnol..

[39] Mertins P., Mani D.R., Ruggles K.V., Gillette M.A., Clauser K.R., Wang P. (2016). Proteogenomics connects somatic mutations to signalling in breast cancer. Nature.

[40] GTEx Consortium (2020). The GTEx Consortium atlas of genetic regulatory effects across human tissues. Science.

[41] Uhlén M., Fagerberg L., Hallström B.M., Lindskog C., Oksvold P., Mardinoglu A. (2015). Proteomics. Tissue-based map of the human proteome. Science.

[42] Ho T.K. (1995). Proceedings of 3rd International Conference on Document Analysis and Recognition (IEEE).

[43] Pedregosa F., Varoquaux G., Gramfort A., Michel V., Thirion B., Grisel O. (2011). Scikit-learn: machine learning in Python. J. Mach. Learn. Res..

[44] Sing T., Sander O., Beerenwinkel N., Lengauer T. (2005). ROCR: visualizing classifier performance in R. Bioinformatics.

[45] Ochoa D., Jonikas M., Lawrence R.T., El Debs B., Selkrig J., Typas A. (2016). An atlas of human kinase regulation. Mol. Syst. Biol..

[46] Akhmedov M., Kedaigle A., Chong R.E., Montemanni R., Bertoni F., Fraenkel E. (2017). PCSF: an R-package for network-based interpretation of high-throughput data. Plos. Comput. Biol..

[47] Cock P.J.A., Antao T., Chang J.T., Chapman B.A., Cox C.J., Dalke A. (2009). Biopython: freely available Python tools for computational molecular biology and bioinformatics. Bioinformatics.

[48] Maglott D., Ostell J., Pruitt K.D., Tatusova T. (2005). Entrez Gene: gene-centered information at NCBI. Nucleic Acids Res..

[49] Sokal R.R. (1958). A statistical method for evaluating systematic relationships : Free Download, Borrow, and Streaming.

[50] Virtanen P., Gommers R., Oliphant T.E., Haberland M., Reddy T., Cournapeau D. (2020). SciPy 1.0: fundamental algorithms for scientific computing in Python. Nat. Methods.

[51] Manning G., Whyte D.B., Martinez R., Hunter T., Sudarsanam S. (2002). The protein kinase complement of the human Genome. Science.

[52] Berginski M.E., Moret N., Liu C., Goldfarb D., Sorger P.K., Gomez S.M. (2021). The Dark Kinase Knowledgebase: an online compendium of knowledge and experimental results of understudied kinases. Nucleic Acids Res..

[53] Chang W., Cheng J., Allaire J., Sievert C., Schloerke B. (2021).

[54] Kuleshov M.V., Jones M.R., Rouillard A.D., Fernandez N.F., Duan Q., Wang Z. (2016). Enrichr: a comprehensive gene set enrichment analysis web server 2016 update. Nucleic Acids Res..

[55] Wu T., Hu E., Xu S., Chen M., Guo P., Dai Z. (2021). clusterProfiler 4.0: a universal enrichment tool for interpreting omics data. Innov. J..

[56] Ashburner M., Ball C.A., Blake J.A., Botstein D., Butler H., Cherry J.M. (2000). Gene ontology: tool for the unification of biology. The Gene Ontology Consortium. Nat. Genet..

[57] Pletscher-Frankild S., Pallejà A., Tsafou K., Binder J.X., Jensen L.J. (2015). DISEASES: text mining and data integration of disease-gene associations. Methods.

[58] Huang R., Grishagin I., Wang Y., Zhao T., Greene J., Obenauer J.C. (2019). The NCATS BioPlanet – an integrated platform for exploring the Universe of cellular signaling pathways for Toxicology, systems biology, and Chemical genomics. Front. Pharmacol..

[59] Krug K., Mertins P., Zhang B., Hornbeck P., Raju R., Ahmad R. (2019). A curated resource for phosphosite-specific Signature analysis. Mol. Cell Proteomics.

[60] Scrucca L., Fop M., Murphy T.B., Raftery A.E. (2016). Mclust 5: clustering, classification and Density estimation using Gaussian finite mixture models. R. J..

[61] Hartigan J.A., Wong M.A. (1979). Algorithm AS 136: a K-means clustering algorithm. J. R. Stat. Soc. Ser. C Appl. Stat..

[62] Rousseeuw P.J. (1987). Silhouettes: a graphical aid to the interpretation and validation of cluster analysis. J. Comput. Appl. Math..

[63] Schwarz G. (1978). Estimating the Dimension of a model. Ann. Stat..

[64] Korotkevich G., Sukhov V., Budin N., Shpak B., Artyomov M.,N., Sergushichev A. (2021).

[65] Qi J., Xu G., Wu X., Lu C., Shen Y., Zhao B. (2023). PELI1 and EGFR cooperate to promote breast cancer metastasis. Oncogenesis.

[66] Saki M., Makino H., Javvadi P., Tomimatsu N., Ding L.-H., Clark J.E. (2017). EGFR mutations Compromise Hypoxia-associated radiation resistance through Impaired Replication Fork-associated DNA damage Repair. Mol. Cancer Res..

[67] Javvadi P., Makino H., Das A.K., Lin Y.F., Chen D.J., Chen B.P. (2012). Threonine 2609 phosphorylation of the DNA-dependent protein kinase is a critical prerequisite for epidermal growth factor receptor-mediated radiation resistance. Mol. Cancer Res..

[68] Kostenko S., Johannessen M., Moens U. (2009). PKA-induced F-actin rearrangement requires phosphorylation of Hsp27 by the MAPKAP kinase MK5. Cell Signal..

[69] Chi Y., Welcker M., Hizli A.A., Posakony J.J., Aebersold R., Clurman B.E. (2008). Identification of CDK2 substrates in human cell lysates. Genome Biol..

[70] Dominguez M., Truemper V., Mota A.C., Brüne B., Namgaladze D. (2022). Impact of ATP-citrate lyase catalytic activity and serine 455 phosphorylation on histone acetylation and inflammatory responses in human monocytic THP-1 cells. Front. Immunol..

[71] Boudeau J., Baas A.F., Deak M., Morrice N.A., Kieloch A., Schutkowski M. (2003). MO25alpha/beta interact with STRADalpha/beta enhancing their ability to bind, activate and localize LKB1 in the cytoplasm. EMBO J..

[72] UniProt Consortium (2025). UniProt: the universal protein knowledgebase in 2025. Nucleic Acids Res..

[73] Cohen P. (2001). The role of protein phosphorylation in human health and disease. The Sir Hans Krebs Medal Lecture. Eur. J. Biochem..

[74] Lussana A., Petsalaki E. (2024). PhosX: data-driven kinase activity inference from phosphoproteomics experiments. bioRxiv.

[75] Halasz M., Kholodenko B.N., Kolch W., Santra T. (2016). Integrating network reconstruction with mechanistic modeling to predict cancer therapies. Sci. Signal..

[76] Terfve C.D.A., Wilkes E.H., Casado P., Cutillas P.R., Saez-Rodriguez J. (2015). Large-scale models of signal propagation in human cells derived from discovery phosphoproteomic data. Nat. Commun..

[77] Thul P.J., Åkesson L., Wiking M., Mahdessian D., Geladaki A., Ait Blal H. (2017). A subcellular map of the human proteome. Science.

[78] Casado P., Rodriguez-Prados J.C., Cosulich S.C., Guichard S., Vanhaesebroeck B., Joel S. (2013). Kinase-substrate enrichment analysis provides insights into the heterogeneity of signaling pathway activation in leukemia cells. Sci. Signal.

[79] Jones D.T., Cozzetto D. (2015). DISOPRED3: precise disordered region predictions with annotated protein-binding activity. Bioinformatics.

[80] Guerois R., Nielsen J.E., Serrano L. (2002). Predicting changes in the stability of proteins and protein complexes: a study of more than 1000 mutations. J. Mol. Biol..

[81] Strumillo M.J., Oplová M., Viéitez C., Ochoa D., Shahraz M., Busby B.P. (2019). Conserved phosphorylation hotspots in eukaryotic protein domain families. Nat. Commun..

[82] Mosca R., Céol A., Aloy P. (2013). Interactome3D: adding structural details to protein networks. Nat. Methods.

[83] Lee B., Richards F.M. (1971). The interpretation of protein structures: estimation of static accessibility. J. Mol. Biol..

[84] Wang M., Weiss M., Simonovic M., Haertinger G., Schrimpf S.P., Hengartner M.O. (2012). PaxDb, a database of protein abundance averages across all three domains of life. Mol. Cell. Proteomics.

[85] Kel A.E., Gössling E., Reuter I., Cheremushkin E., Kel-Margoulis O.V., Wingender E. (2003). MATCH: a tool for searching transcription factor binding sites in DNA sequences. Nucleic Acids Res..

[86] Pollastri G., Baldi P., Fariselli P., Casadio R. (2002). Prediction of coordination number and relative solvent accessibility in proteins. Proteins.

[87] Dana J.M., Gutmanas A., Tyagi N., Qi G., O’Donovan C., Martin M. (2018). SIFTS: updated Structure Integration with Function, Taxonomy and Sequences resource allows 40-fold increase in coverage of structure-based annotations for proteins. Nucleic Acids Res..

[88] Bateman A., Martin M.J., O’Donovan C., Magrane M., Alpi E., Antunes R., The UniProt Consortium (2016). UniProt: the universal protein knowledgebase. Nucleic Acids Res..

[89] Dinkel H., Van Roey K., Michael S., Kumar M., Uyar B., Altenberg B. (2016). ELM 2016--data update and new functionality of the eukaryotic linear motif resource. Nucleic Acids Res..

[90] Hopf T.A., Ingraham J.B., Poelwijk F.J., Schssärfe C.P.I., Springer M., Sander C. (2017). Mutation effects predicted from sequence co-variation. Nat. Biotechnol..

