# Supplementary material for: Data-Driven Extraction of Human Kinase-Substrate Relationships From Omics Datasets
Source: Mol Cell Proteomics. 2025 May 15;24(8):100994. doi: 10.1016/j.mcpro.2025.100994 (PMC12359690; doi:10.1016/j.mcpro.2025.100994)
Supplement: Supporting Information 1 [file mmc1.pdf]

Data-driven extraction of human kinase-substrate relationships from omics datasets

Benjamin Dominik Maier[1#], Borgthor Petursson[1#], Alessandro Lussana[1#] and Evangelia Petsalaki[1,*]

[1]European Molecular Biology Laboratory, European Bioinformatics Institute (EMBL-EBI), Wellcome Genome Campus, Hinxton, Cambridgeshire, CB10 1SD, United Kingdom

*Correspondence should be addressed to Evangelia Petsalaki, petsalaki@ebi.ac.uk

[#]These authors contributed equally

Running Title: Prediction of human kinase-substrate relationships

Supplementary information file table of contents

Supplemental Figures 1-6

Supplemental Table 1

Supplemental Table 3

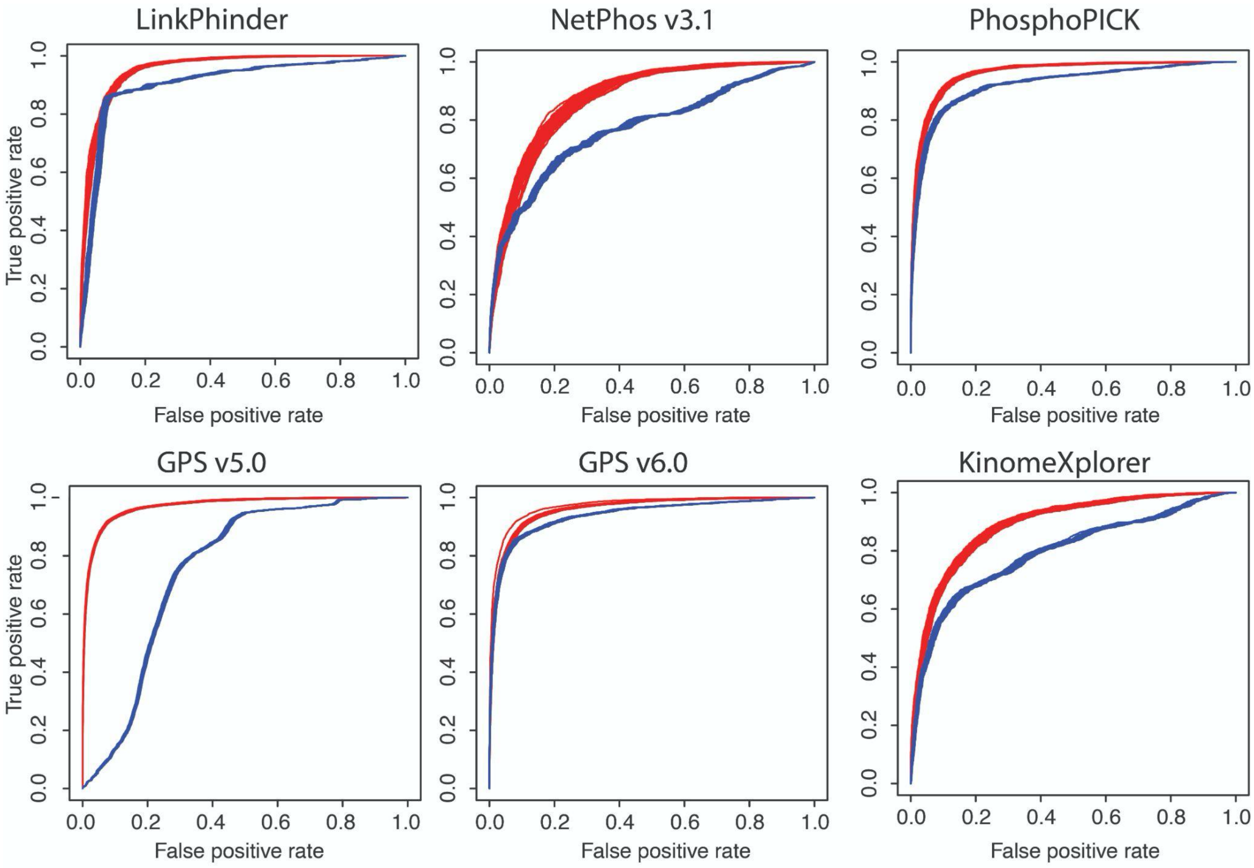

Supplemental Figure 1. Comparison of performance of SELPHI$_{2.0}$ (red) with 6 state-of-the-art methods (blue). Comparison was done using 100 different positive/negative training set combinations as described in Experimental Procedures.

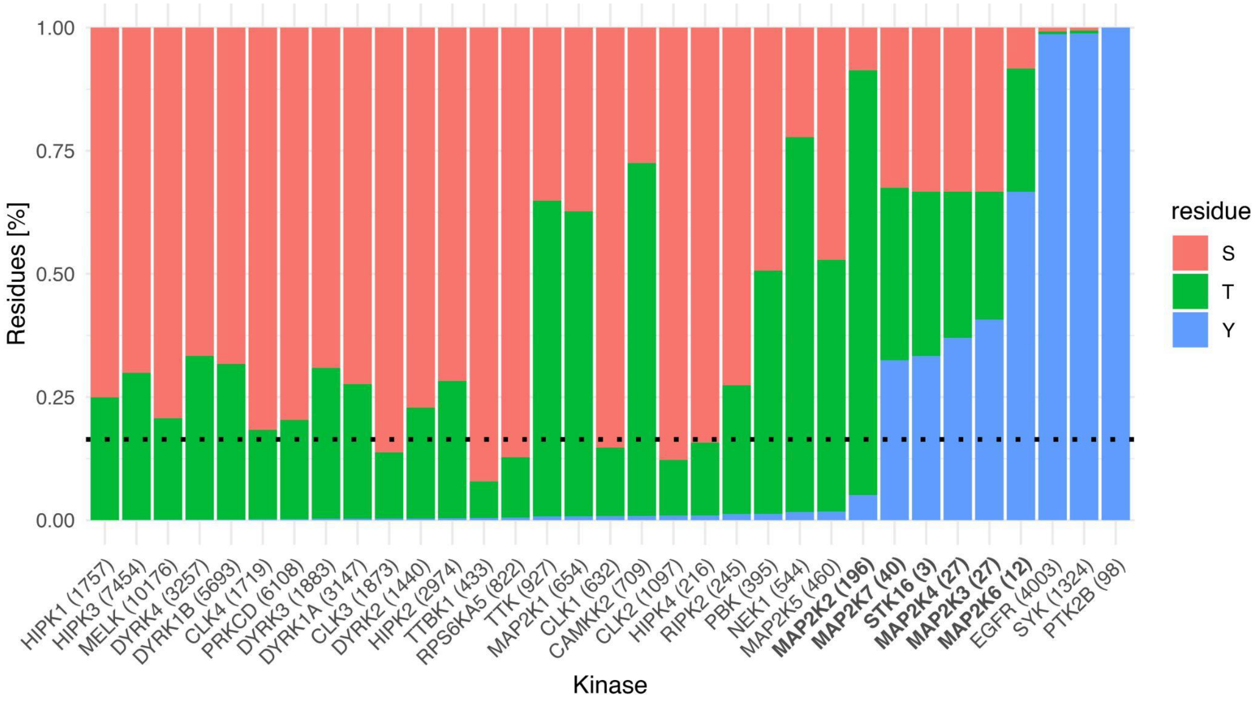

Supplemental Figure 2. Distribution of phosphosite type predictions for the kinases annotated as dual specificity (see Experimental Procedures). Bold kinases indicate those that we predict are indeed dual specificity. The dotted line indicates the percentage of phosphosites that are tyrosine sites in the entire dataset.

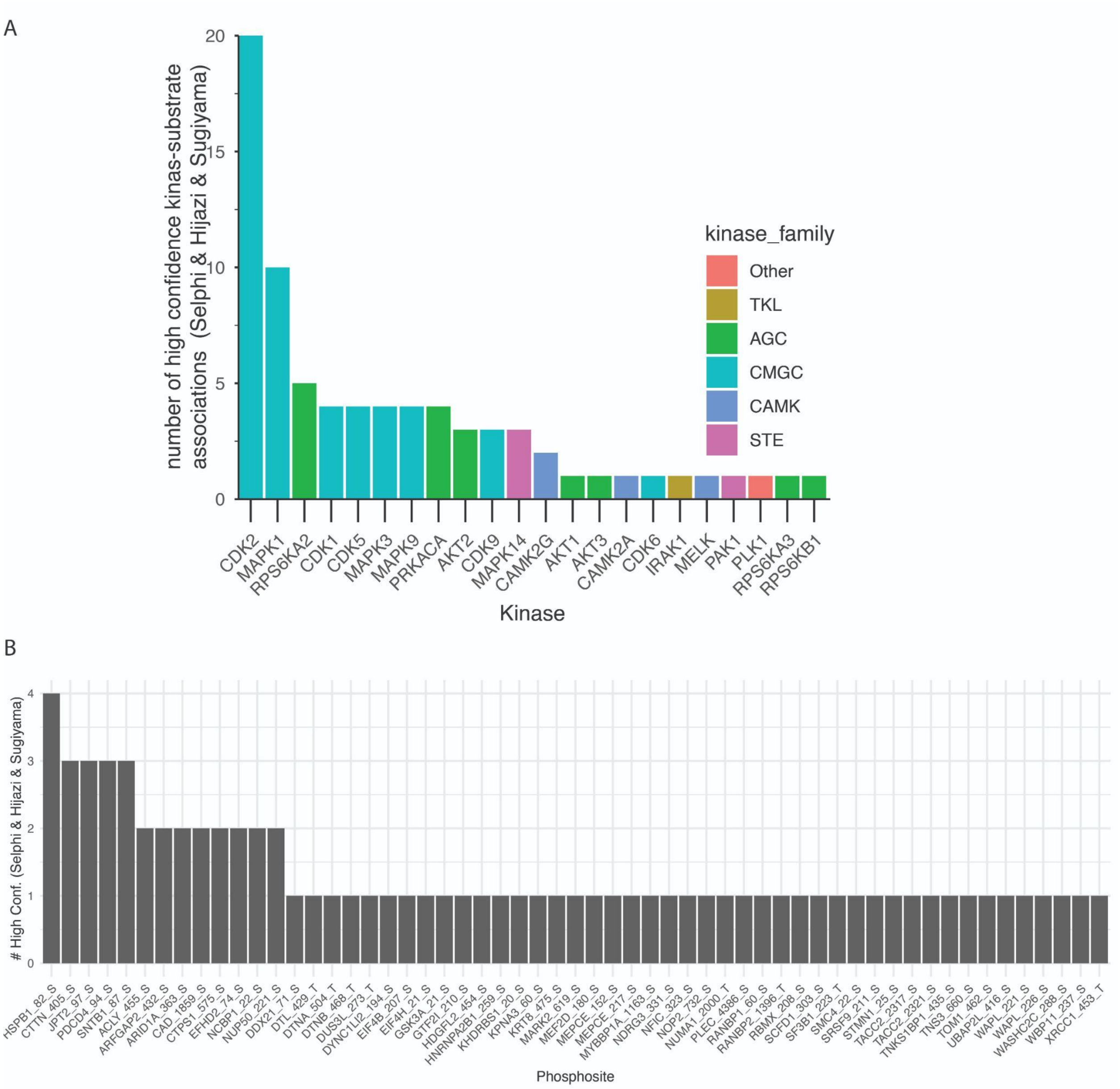

Supplemental Figure 3. Distribution of high confidence hits per kinase (A) and substrate phosphosite (B)

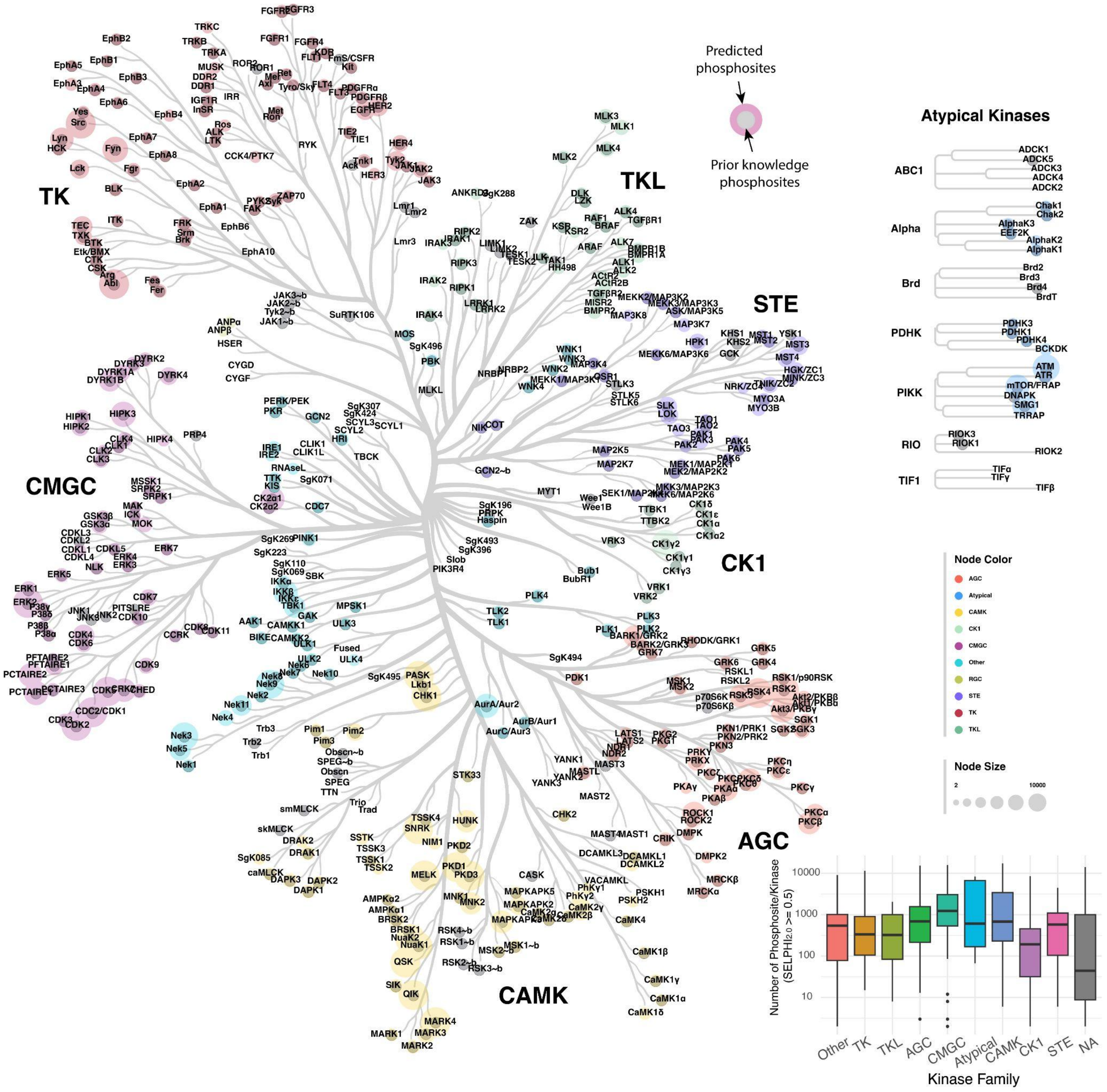

Supplemental Figure 4. Distribution of known and predicted substrates for all kinases mapped on the kinase phylogenetic tree. Paired with Figure 3A, made larger to allow annotation of kinase names. Outer-colored circle indicates predicted substrates, inner gray circle indicates previously known substrates. Inset in bottom right of the figure shows the distribution of substrates predicted per kinase family.

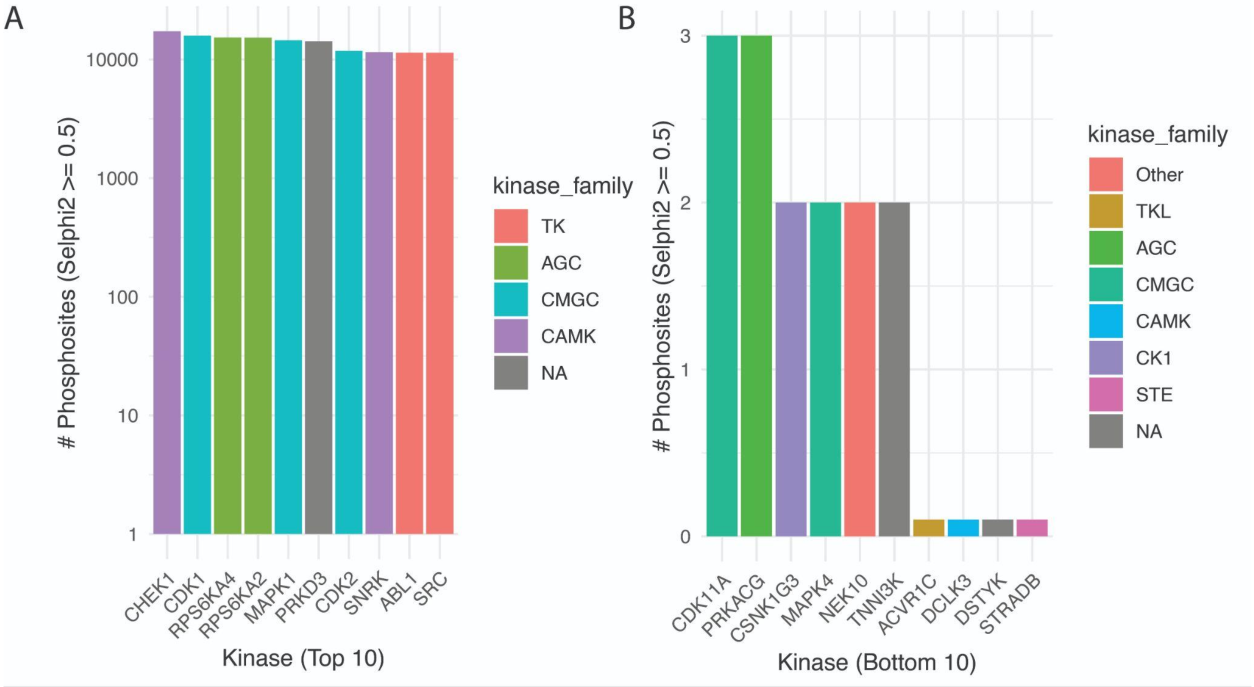

Supplemental Figure 5. Top (A) and Bottom (B) 10 kinases in terms of numbers of phosphosites predicted as their targets.

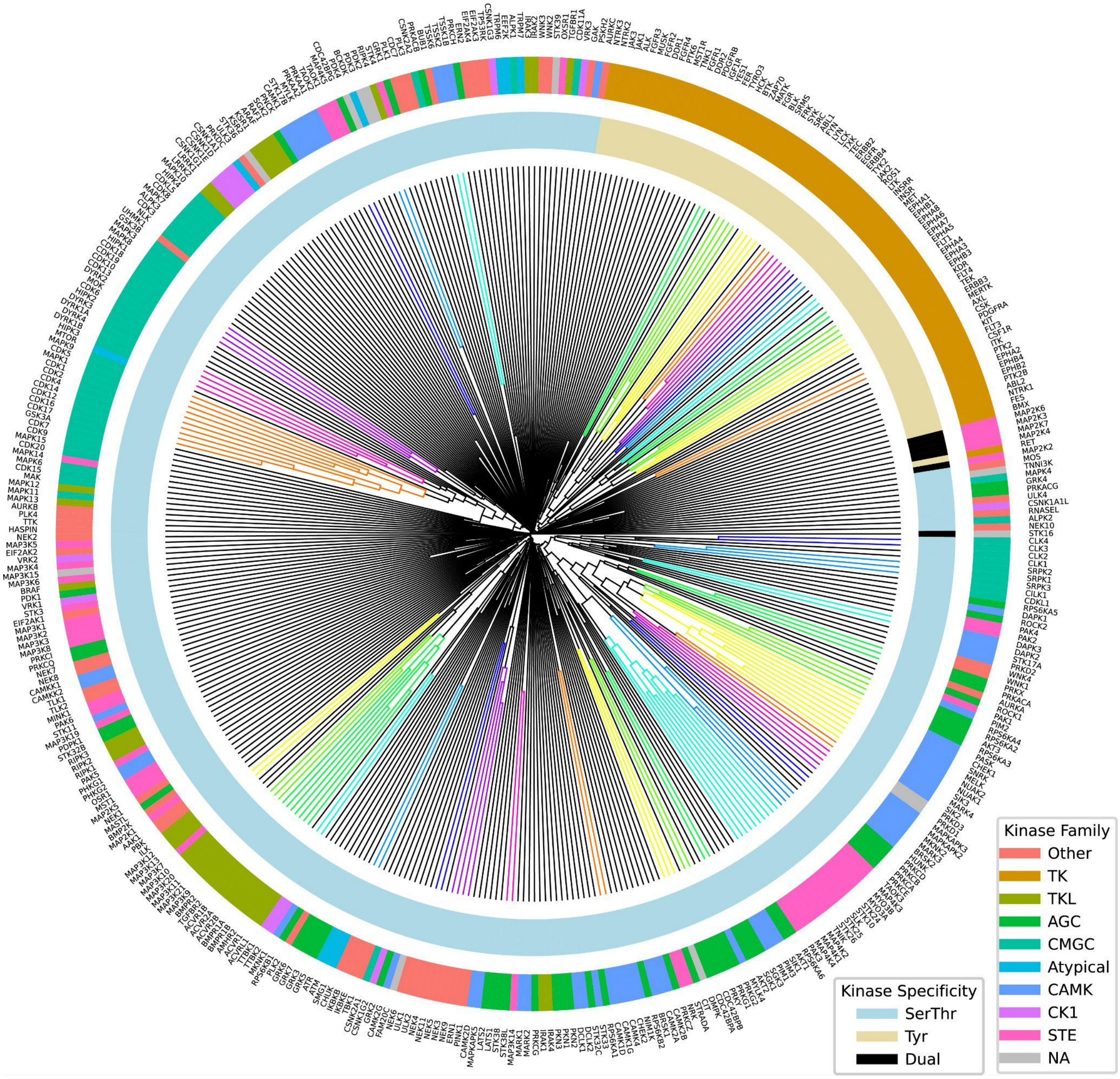

Supplemental Figure 6. Kinases clustered based on the overlap of their predicted substrates (distance=1-Jaccard index). Outer circle is colored by kinase family, inner circle by kinase specificity and the colors on the tree are provided to highlight those clusters of kinases that had a distance < 0.7.

Supplemental Table 1. Features used in the development of the machine learning model to predict human kinase-substrate relationships.

Name of feature	Description	Imputation
Residue	Indicator of phospho acceptor residue: S/T/Y	No missing values
PWM score	Score generated by fitting phosphosites to PWMs described in previous publication(28).	0
Coreg_293	Spearman's correlation between estimated kinase activities and target phosphosite's phosphorylation(39). Kinase activities were estimated with the KSEA algorithm(45)	0
Functional_score	Score indicating the probability of phosphosite to be functional(38, 45).	No missing values
GTEx	Co-expression between kinase and putative substrate genes across tissues(40). Co-expression was calculated using Spearman's correlation.	0
RNA_tissue	Co-expression between kinase and putative substrate gene across tissues(41). Co-expression was calculated using Spearman's correlation	0
RNA_cell	Co-expression between kinase and putative substrate gene across cell lines(77). Co-expression was calculated using Spearman's correlation	0
Kinase_selectivity	Skewness of kinase expression distribution across different tissues(41). Skewness was calculated by using the e1071 R library to calculate the skewness (https://CRAN.R-project.org/package=e1071).	0

Substrate_selectivity	Skewness of substrate gene expression distribution across different tissues(41). Skewness was calculated by using the e1071 R library to calculate the skewness (https://CRAN.R-project.org/package=e1071).	0
NTERA2_coreg	Spearman's correlation between estimated kinase activities and target phosphosite's phosphorylation across 63 inhibitor conditions in NTERA2 cell line(33). Kinase activities were estimated with the KSEA algorithm(78).	0
MCF7_coreg	Spearman's Correlation between estimated kinase activities and target phosphosite's phosphorylation across 63 inhibitor conditions in MCF7 cell line(33). Kinase activities were estimated with the KSEA algorithm(78).	0
HL60_coreg	Spearman's correlation between estimated kinase activities and target phosphosite's phosphorylation across 63 inhibitor conditions in HL60 cell line(33). Kinase activities were estimated with the KSEA algorithm(78).	0
Is_DISOPRED	Indicates if phosphosite is disordered (DISOPRED score(79) > 0.5). This feature was downloaded from previous publication by Ochoa and colleagues(38).	Phosphosites with missing values are assumed to be disordered
DISOPRED score	DISOPRED score of target phosphosite(79). This feature was downloaded from previous publication by Ochoa and colleagues(38).	Median imputation
Exp3d_ala_ddG_effect	Discretized changes in Gibb's energy upon mutation from original residue to alanine. Measured by FoldX v4(80). This feature was downloaded from previous publication by Ochoa and colleagues(38).	Missing data set to unknown

Exp3d_acid_ddG_effect	Discretized average changes in Gibb's energy upon mutation from original residue to acidic residue Measured by FoldX v4(80).. This feature was downloaded from previous publication by Ochoa and colleagues(38).	Missing data set to unknown
Log_10_of_hotspot_pval_min	Quantification of the prevalence of phosphorylation events within the structural region. The p-value indicates the enrichment of phosphosites within the region in question(81). This feature was downloaded from previous publication by Ochoa and colleagues(38).	0
Is_hotspot	Indicates If the phosphosite has assigned hotspot enrichment value of <3.36e-07 and is present in more than 10 MS data sets (81). This feature was downloaded from previous publication by Ochoa and colleagues(38).	Missing values set to FALSE
Is_Interface	Experimentally resolved or modelled interaction interfaces were obtained from Interactome3d(82). NACCESS(83) was used to calculate relative solvent accessibility of atoms. In cases where the relative solvent accessibility differed between interacting and non-interacting form, the residue was considered to be found on the interface. This feature was downloaded from a previous publication by Ochoa and colleagues(38).	Missing values are set to FALSE
Adj_ptms w21	Number of phosphosites within +/- 10 residue window on either side of the target phosphosite. This feature was downloaded from a previous publication by Ochoa and colleagues(38).	Zero
Netpho_max_all	Highest posterior probability obtained from all NetPHOREST v2.1(19) models. This feature was downloaded from a previous publication by Ochoa and colleagues(38).	Median imputation

Netpho_max_KIN	Highest posterior probability obtained from all kinase models found in NetPHOREST v2.1(19). This feature was downloaded from a previous publication by Ochoa and colleagues(38).	Median imputation
Paxdb_abundance_log10	Consensus protein abundance downloaded from the PaxDb(84) database. This feature was downloaded from a previous publication by Ochoa and colleagues(38).	Median imputation
W0_mya	Age of the target phosphosite. This feature was downloaded from a previous publication by Ochoa and colleagues(38).	0
W3_mya	Age of the +/- 3 residue region surrounding the target phosphosite. This feature was downloaded from a previous publication by Ochoa and colleagues(38).	0
Quant_top1	Number of times phosphosite was found within the top 1% of regulated sites across 435 conditions(45). This feature was downloaded from a previous publication by Ochoa and colleagues(38).	0
Quant_top5	Number of times phosphosite was found within the top 5% of regulated sites across 435 conditions(45). This feature was downloaded from a previous publication by Ochoa and colleagues(38).	0
PWM_max_mss	The best fit to one of the PSSMs of 143 different kinases known to have at least 10 known substrates as measured by the MATCH algorithm(85). This feature was downloaded from a previous publication by Ochoa and colleagues(38).	Median imputation
ACCpro	Prediction of solvent accessibility with ACCpro(86). This feature was downloaded from a previous publication by Ochoa and colleagues(38).	Missing values set to unknown
SSpro	Prediction of secondary structure with SSpro(86). This feature was downloaded from a previous publication by Ochoa and colleagues(38).	Missing values set to unknown
SSpro8	Indicates the class of secondary structure as predicted by SSpro8(86). This feature was downloaded from a previous publication by Ochoa and colleagues(38).	Missing values set to unknown

SIFT_min_score	Conservation using SIFT score as a proxy(87). This feature contains the minimum score across all variants. This feature was downloaded from a previous publication by Ochoa and colleagues(38).	Median imputation
SIFT_mean_score	Conservation using SIFT score as a proxy(87). This feature contains the mean score across all variants. This feature was downloaded from a previous publication by Ochoa and colleagues(38).	Median imputation
SIFT _ala_score	Conservation using SIFT(87) score as a proxy. This feature contains the score of alanine variants. This feature was downloaded from a previous publication by Ochoa and colleagues(38).	Median imputation
SIFT_acid_score	Conservation using SIFT(87) score as a proxy. This feature contains the average score of variants leading to negative charge. This feature was downloaded from a previous publication by Ochoa and colleagues(38).	Median imputation
IsProteinDomain	This feature indicates if the target phosphosite is found within a protein domain(88). This feature was downloaded from a previous publication by Ochoa and colleagues(38).	Missing values set to FALSE
IsProteinKinaseDomain	This feature indicates if the target phosphosite is found within a kinase domain(88). This feature was downloaded from a previous publication by Ochoa and colleagues(38).	Missing values set to FALSE
IsUniprotRegion	This feature indicates if the target phosphosite is found within any other curated motif(88). This feature was downloaded from a previous publication by Ochoa and colleagues(38).	Missing values set to FALSE
IsCytoplasmic	This feature indicates if the target phosphosite is found within a cytosolic region of a transmembrane protein(88). This feature was downloaded from a previous publication by Ochoa and colleagues(38).	Missing values set to FALSE
IsMotif	This feature indicates whether the target phosphosite is found within any other curated motif(88). This feature was downloaded from a previous publication by Ochoa and colleagues(38).	Missing values set to FALSE

IsELMLinearMotif	This feature indicates if the flanking region of the phosphosite is listed under linear motifs (CLV, DEG, DOC, MOD, TRG, and/or kinase motif)) in ELM(89). This feature was downloaded from a previous publication by Ochoa and colleagues(38).	Missing values set to FALSE
IsEV_ala_prediction_epistatic5	This feature indicates if alanine mutation leads to epistatic effects as calculated with the EVmutation algorithm(90). This feature was downloaded from a previous publication by Ochoa and colleagues(38).	Missing values set to FALSE
IsKinaseCoreg	This feature indicates if the target phosphosite is coregulated with a kinase. Derived from kinase activities estimated in previous publication. This feature was downloaded from a previous publication by Ochoa and colleagues(38).	Missing values set to FALSE
serThrPssmScore	The transformed PSSM score between the given kinase-phosphosite pair. The raw PSSM score(27) is transformed between 0 and 1 based on the quantile it falls in, considering a kinase-specific background distribution of proteome-wide raw PSSM scores. These background scores are computed on a set of more than 200,000 human phosphosites annotated in the PhosphositePlus database(37) (September 2023). Phosphosites with raw PSSM score equal to 0 are discarded, and the remaining are used to determine the values of the 10,000-quantiles of the raw PSSM score distribution.	0
tyrPssmScore	This feature is computed exactly as serThrPssmScore, but using the PSSMs sourced from(26)	0
Excluded Features		
IsUniprotRepeat	*This feature indicates if the target phosphosite is found within a repeated motif(88). This feature was downloaded from a previous publication by Ochoa and colleagues(38).*	*Missing values set to FALSE*
IsUniprotZnFinger	*This feature indicates if the target phosphosite is found within a zinc finger(88). This feature was downloaded from a previous publication by Ochoa and colleagues(38).*	*Missing values set to FALSE*

IsUniprotCompBias	*This feature indicates if the target phosphosite is found within a compositionally biased region(88). This feature was downloaded from a previous publication by Ochoa and colleagues(38).*	*Missing values set to FALSE*

Supplementary table 3. Overview over the number of kinase substrate relationships, kinases and the size of the positive set as well as the overlap with Sugiyama et al.

Method	Number of supported kinases	Number of kin-substrates shared with $SELPHI_{2.0}$	Number of kin-substrates shared with $SELPHI_{2.0}$ and predicted by Sugiyama et al.
PhosphoPICK	107	7,006,604	17,060
GPS v5.0	479	18,319,104	65,141
GPS v6.0	349	25,173,556	71,882
NetPhos v3.1	17	5,528,807	13,116
LinkPhinder	327	1,343,230	4,568
KinomeXplorer	193	7,750,644	30,645
